# Radiological evaluation of fusion patterns after Lateral Lumbar Interbody fusion with 3D-printed porous titanium cages vs. conventional titanium cages

**DOI:** 10.3389/fsurg.2024.1446792

**Published:** 2024-10-15

**Authors:** Calogero Velluto, Gregory Mundis, Laura Scaramuzzo, Andrea Perna, Giacomo Capece, Andrea Cruciani, Michele Inverso, Maria Ilaria Borruto, Luca Proietti

**Affiliations:** ^1^ Catholic University of the Sacred Heart, Rome, Italy; ^2^ Department of Aging, Orthopaedic and Rheumatological Sciences, Fondazione Policlinico Universitario Agostino Gemelli IRCCS, Rome, Italy; ^3^ Division of Spine Surgery, Department of Orthopedics, Scripps Clinic, San Diego, CA, United States; ^4^ Department of Orthopaedics and Traumatology, Fondazione Casa Sollievo della Sofferenza IRCCS, San Giovanni Rotondo, Italy

**Keywords:** fusion, instrumentation, LLIF, minimally invasive spine surgery, porous titanium, subsidence

## Abstract

**Introduction:**

The assessment of segmental fusion after Lateral Lumbar Interbody fusion (LLIF) using 3D-printed porous titanium cage is still not well studied. Various criteria, such as the presence of bone bridges (BB) between adjacent vertebrae, serve as indicators for anterior fusion. However, limited radiological studies have investigated zygapophyseal joints (ZJ) status following LLIF with porous titanium cages vs. conventional titanium threaded cages. The porous design of the latest titanium intervertebral cages is thought to enhance the bone-to-implant fusion rate. This radiological study aimed to compare the fusion patterns post-LLIF using 3D-printed porous titanium cages against those using threaded titanium cages. This radiological study aimed to compare the fusion patterns after LLIF using 3D-printed porous titanium cages against those using threaded titanium cages.

**Material and methods:**

This retrospective, single-center radiological study involved 135 patients who underwent LLIF and posterior percutaneous screw fixation for degenerative spondylolisthesis. The study included 51 patients (Group A) with the novel porous titanium cages and 84 patients (Group B) with conventional threaded titanium cages. Inclusion criteria mandated complete radiological data and a minimum follow-up period of 24 months. The study evaluated intervertebral bone bridges (BB) for anterior fusion and zygapophyseal joints (ZJ) ankylotic degeneration, based on Pathria et al., as evidence of posterior fusion and segmental immobilization.

**Results:**

Two years after surgery, intervertebral BB were identified in 83 segments (94.31%) in Group A and in 87 segments (88.77%) in Group B. ZJ Pathria grade I was observed in 2 segments (2.27%) of Group A and in 4 segments (4.08%) of Group B. Grade II was seen in 5 segments (5.68%) of Group A and in 6 segments (6.12%) of Group B. Posterior fusion, classified as grade III, was found in 81 segments (92.04%) of Group A and 88 segments (89.79%) of Group B. Subsidence incidence was 5.88% (3 segments) for the novel cage and 9.88% (8 segments) for the conventional cage.

**Conclusions:**

The architecture of porous titanium cages offers a promising solution for increasing bone ingrowth and bridging space, supporting successful spinal fusion while minimizing the risk of subsidence.

## 1 Introduction

Minimally invasive surgery (MIS) has significantly transformed spinal surgery, with Lateral Lumbar Interbody Fusion (LLIF) emerging as a reliable and effective method for addressing various spinal conditions, including degenerative disc disease, spondylolisthesis, degenerative scoliosis, and adjacent segment degeneration (1). LLIF offers notable advantages such as less tissue disruption, reduced blood loss, and shorter hospital stays compared to traditional open techniques (2). However, evaluating the success of LLIF, particularly in terms of segmental fusion, remains complex due to factors such as cage design, materials, and patient-specific variables. While LLIF has shown similar postoperative outcomes to conventional lumbar interbody fusions, segmental fusion evaluation continues to pose challenges (3). Standard criteria for assessing fusion include identifying bone bridges (BB) between vertebrae, indicative of anterior fusion. Nevertheless, comprehensive studies on zygapophyseal joints (ZJ) status after LLIF, especially with the use of 3D-printed porous titanium cages, are lacking. The presence of intervertebral fusion through BB is a recognized criterion, underscoring the importance of anterior fusion to prevent micromovements, mechanical stress, and potential failures. The significance of meticulous disc space preparation, instrumentation, cage design, and material properties cannot be overstated for successful fusion. Recent innovations in porous titanium cages have shown potential in enhancing bone-to-implant fusion rates, reducing subsidence, and ensuring overall success in spinal fusion procedures. Research supports that porous materials mimicking cancellous bone structure can lower stress shielding and subsidence at the bone-hardware interface (4). This scientific progress has led to the creation of advanced porous 3D-printed titanium interbody cages, featuring intricate internal architectures and textured surfaces that promote osteoblastic activity and integration into bone. Despite these advances, there remains a gap in understanding the detailed differences in fusion patterns between 3D-printed porous titanium cages and conventional threaded cages in the context of LLIF. The primary goal of this radiological study was to assess segmental fusion outcomes in patients undergoing LLIF, comparing the efficacy of 3D-printed porous titanium cages with traditional threaded cages. This study includes an evaluation of intervertebral bone bridges, zygapophyseal joint status, and the incidence of subsidence of the implanted cages. Additionally, the findings aim to provide valuable insights into the evolving techniques of LLIF, highlighting the potential advantages of porous titanium cages in achieving successful spinal fusion and minimizing the risk of implant subsidence.

## 2 Material and methods

### 2.1 Study design and settings

This study is a retrospective investigation conducted at a single center. Data were gathered from the institutional Picture Archiving and Communication System (PACS) spanning from January 2018 to December 2020.

### 2.2 Inclusion and exclusion criteria

The study selected patients from the institutional database who had undergone LLIF (Lateral Lumbar Interbody Fusion) and posterior percutaneous screw fixation for degenerative lumbar spondylolisthesis. Eligibility required complete clinical and radiological data, pre-collected informed consent for scientific purposes, and a minimum follow-up period of 24 months.

The exclusion criteria included:

1. Preoperative bone density with a *t*-score < −2.0, as determined by Dual Energy x-Ray Absorptiometry (DEXA);
2. Ankylosing spondylitis or diffuse idiopathic skeletal hyperostosis;
3. Prior lumbar spinal surgery;
4. Abnormal pre- or postoperative spinopelvic parameters (5, 6);
5. Postoperative infection;
6. Neoplastic diseases;
7. Intraoperative evidence of cage subsidence.

### 2.3 Surgical technique

All patients initially underwent LLIF, following the method described by Ozgur et al. (7), utilizing the XLIF system (Nuvasive, San Diego, CA, USA). A meticulous discectomy was performed, avoiding endplate violations. The cages implanted were either Titanium threaded or 3D-printed porous titanium (XLIF Modulus, Nuvasive, San Diego, CA, USA), filled with graft material (Synthetic bone Nuvasive AttraX Putty, 25 × 9 × 13.5 mm, 6 cc). Posterior percutaneous fixation with titanium pedicle screws and rods (Reline, Nuvasive, San Diego, CA, USA) was performed in a second stage, with the patient in a prone position, ensuring the Zygapophyseal Joint (ZJ) was not violated.

### 2.4 Radiological outcomes

Preoperative and 24-month postoperative CT scans and x-ray images were reviewed using a dedicated workstation (Advantage Windows Workstation; GE Medical Systems, Milwaukee, USA). Interbody BB inside or around the cage, characterized by trabeculae linking the cancellous bone of the two vertebrae without the endplate cortical rim, were considered indicative of segmental fusion (8). ZJ were evaluated preoperatively and 24 months post-surgery, with their fusion patterns classified according to Pathria et al. (9). They were deemed fused when Pathria grade III was associated with BB interbody fusion and non-fused for lower Pathria grades. Posterior fusion due to ankylotic degeneration was used as a criterion for segmental immobilization (10). CT images were independently assessed by three authors and a senior spinal surgeon (L.P.).

### 2.5 Clinical outcomes

Clinical status was assessed preoperatively and 24 months post-surgery using a ten-point visual analogue scale (VAS) for leg (VAS-l) and back (VAS-b) pain, and the Oswestry Disability Index (ODI) score. Intraoperative and postoperative complications were recorded.

### 2.6 Statistical analysis

Data are presented as means and standard deviations (SD). Normality was tested. Categorical variables were compared using the two-tailed Fisher exact test, while continuous variables were compared using *T*-tests. Interrater reliability (IRR) between the three evaluators was calculated using Fleiss’ kappa statistic. A significance level of 0.05 was used. SPPS ± statistical calculation software (SOSS Inc, Chicago, IL) was utilized for data analysis.

## 3 Results

### 3.1 Participants

From January 2018 to December 2020, the study included 135 patients (54 males and 81 females) who underwent single- or multi-level minimally invasive trans-psoas LLIF using either novel 3D-printed porous titanium cages or titanium threaded cages. The average age was 66 ± 6.7 years, the average body mass index (BMI) was 28.33, and the average follow-up period was 32 months (ranging from 24 to 41 months). Patient data are detailed in Table 1.

**Table 1:** Patients data.

Demographical	
Age	66 ± 6.7
Sex (F/M)^a^	81 (60%)/54 (40%)
BMI	28.33 ± 1.3
Diabetes^a^	29 (21.48%)
Smokers^a^	34 (25.18%)
Instrumentated level^a^	
L1–2	9 (4.84%)
L2–3	31 (17%)
L3–4	81 (43.54%)
L4–5	65 (34.94%)
Mean follow-up (months)	32 ± 3.5

Values are reported as mean ± standard deviation.

^a^ Values are reported as number of patients (percentage).

### 3.2 Surgical data

In total, 186 cages were implanted among the 135 patients. This included 88 (47.3%) 3D-printed porous titanium cages and 98 (52.68%) conventional titanium cages. The left side was used for the lateral stage in 112 patients (82.96%). The average duration of ileal-psoas muscle retraction was 21 min (±6.3, range 18–29 min). The mean estimated intraoperative blood loss was 60 ml (±48.5 ml, ranging from 15 to 130 ml), with no cases requiring postoperative blood transfusions. There were 21 cases (15.55%) of transient dysesthesia on the anterior-medial surface of the thigh (ipsilateral to the surgical approach), 7 cases (5.18%) of postoperative paralytic ileus (resolved spontaneously within 3 days), and 4 cases (3%) of abdominal wall twitching. No cases required reoperation.

### 3.3 Radiological findings

**Figure 1:**
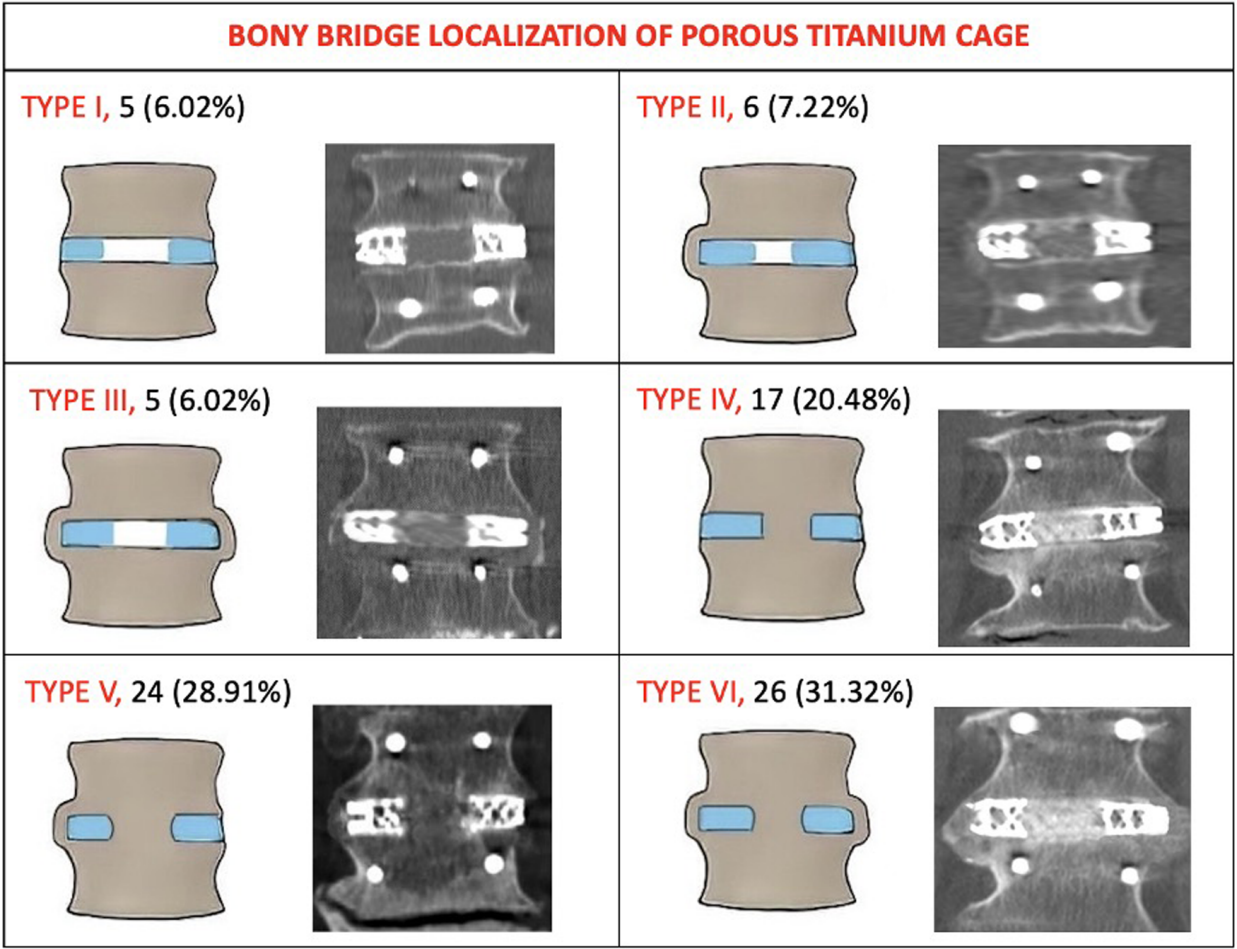
The topography of intervertebral bone bridges of Group A (Porous 3D printed titanium cage). Type I: no bone bridges (no fusion). Type II: bone bridges monolateral extra cage. Type III: bone bridges bilateral extra cage. Type IV: bone bridges inside the internal spaces of the cage. Type V: bone bridges inside the internal spaces of the cage and on one side extra cage. Type VI: bone bridges inside the internal spaces of the cage and on both sides extra cage. Lateral view pattern fusion: Subtype A: absence of a bone bridge either anteriorly or posteriorly. Subtype B: presence of a bone bridge only anteriorly to the cage. Subtype C: presence of bone bridge only posteriorly to the cage. Subtype D: presence of bone bridges both anteriorly and posteriorly to the cage.

At 24 months, computed tomography demonstrated intervertebral BB solid fusion inside or around the cage, characterized by trabeculae linking the cancellous bone of the two vertebrae, in 172 segments (92.97%). In 83 segments (94.31%) treated with the novel porous 3D-printed titanium cage (Group A), anterior fusion was observed. In patients treated with the conventional cage (Group B), anterior fusion was reported in 87 segments (88.77%). Among Group B patients, one or more BB were reported inside the cage in every anteriorly fused segment, and in 51 segments (52.04%), there was also evidence of at least one BB outside the implant. The topographical fusion patterns are summarized in Figure 1 for Group A. ZJ were evaluated preoperatively and 24 months post-surgery, and their fusion patterns were classified according to Pathria et al. At the 24-month radiological follow-up, Pathria grade I was seen in 2 segments (2.27%) of Group A patients and in 4 segments (4.08%) of Group B patients. Grade II was reported in 5 segments (5.68%) of Group A patients and in 6 segments (6.12%) of Group B patients. Grade III was observed in 81 segments (92.04%) of Group A patients and in 88 segments (89.79%) of Group B patients. The IRR was calculated using Fleiss’ kappa (0.891, 96% CI: 0.513–0.769). Spinopelvic parameters measured preoperatively and at the 24-month radiological follow-up for all 135 patients were: Lumbar Lordosis (LL) 41.8° (±8.1)–47.8° (±9.3) (*p* = 0.08); Sacral Slope (SS) 30.5° (±6.4)–31.1° (±6.7) (*p* = 0.61); Pelvic Tilt (PT) 20.7° (±4.9)–19.2° (±5.1) (*p* = 0.36). The mean disc angle changed from 2.8° (±1.7) to 7.2° (±2.1) (*p* = 0.00342). There were no statistically significant differences in spinopelvic parameter modifications between immobilized and non-immobilized segments, or between fused, partially fused, and non-fused levels. Finally, the subsidence rate was 5.88% (3 segments) for the novel cage and 9.88% (8 segments) for the conventional cage, with a statistically significant difference (*p* < 0.004).

### 3.4 Fusion patterns analysis

Two years post-surgery, 2 segments (2.27%) in Group A and 3 segments (3.06%) in Group B showed neither anterior nor posterior fusion (*p* = 0.12). Anterior interbody fusion only was achieved in 17 patients (19.32%) in Group A and 18 patients (18.37%) in Group B, showing a statistically significant difference (*p* < 0.04). Posterior fusion only was observed in 5 patients (5.68%) in Group A and 8 patients (8.16%) in Group B (*p* = 0.23). Concurrent anterior and posterior fusions were found in 81 patients (92.04%) in Group A and 88 patients (89.79%) in Group B, also showing a statistically significant difference (*p* = 0.02).

### 3.5 Clinical outcomes

The mean VAS-b score improved from 7.9 (±1.5) preoperatively to 2.3 (±1.7) at 24 months post-surgery in the fused group (*p* = 0.00117), from 8.1 (±0.7) to 2.9 (±1.8) in the partially fused group (*p* = 0.0020), and from 8.2 (±1.5) to 4.9 (±2.1) in the non-fused group (*p* = 0.0131). At the last follow-up, the mean VAS-b score in the non-fused group was significantly higher than in the fused (*p* = 0.0025) and partially fused (*p* = 0.0042) groups. The mean preoperative VAS-l score decreased from 6.7 (±1.4) to 1.8 (±1.3) at 24 months in the fused group (*p* = 0.0039), from 7.6 (±1.5) to 1.8 (±1.6) in the partially fused group (*p* = 0.0013), and from 7.6 (±1.9) to 3.5 (±1.9) in the non-fused group (*p* = 0.0045). No statistically significant differences were observed between the three groups preoperatively or at the last follow-up measurements. The mean ODI score changed from 41% (±7) preoperatively to 17% (±5) at 24 months in the fused group (*p* = 0.0001), from 43% (±11) to 21% (±7) in the partially fused group (*p* = 0.0031), and from 50% (±8) to 42% (±12) in the non-fused group (*p* = 0.028). The mean ODI score was significantly higher in the non-fused group compared to the fused (*p* < 0.001) and partially fused (*p* = 0.0067) groups. Among immobilized and non-immobilized patients, the mean VAS-b scores were 8.3 (±1.7) and 8.3 (±1.2) preoperatively (*p* = 0.38), then 2.7 (±1.4) and 4.8 (±1.9) at 24 months (*p* = 0.0038); the mean VAS-l scores were 6.8 (±1.7) and 7.1 (±1.9) preoperatively (*p* = 0.179), then 3 (±1.4) and 4.7 (±1.4) at 24 months (*p* < 0.001); the mean ODI scores were 42% (±12) and 46% (±10) preoperatively (*p* = 0.654), then 46% (±10) and 35% (±9) at 24 months (*p* = 0.0169). No differences were found in clinical outcomes (VAS-l, VAS-b, ODI) when comparing fused and immobilized patients at the last follow-up. Clinical data are summarized in Table 2.

**Table 2:** Clinical outcome, trend overtime of patients.

	No. of pt	Pre-op VAS-b	24 m VAS-b	*p* value	Pre-op VAS-l	24 m VAS-l	*p* value	Pre-op ODI%	24 m ODI%	*p* value
Fused (F)	65	7.9 ± 1.5	2.3 ± 1.7	0.00117	6.7 ± 1.4	1.8 ± 1.3	0.0039	41 ± 7	17 ± 5	0.0001
Partial fused (PF)	53	8.1 ± 0.7	2.9 ± 1.8	0.0020	7.6 ± 1.5	1.8 ± 1.6	0.0013	43 ± 11	21 ± 7	0.0031
Non fused (NF)	17	8.2 ± 1.5	4.9 ± 2.1*	0.0131	7.6 ± 1.9	3.5 ± 1.9	0.0045	50 ± 8	42 ± 12*	0.028
Immobilized (I)	117	8.3 ± 1.7	2.7 ± 1.3	0.0038	6.8 ± 1.7	3 ± 1.4	0.00479	42 ± 12	20 ± 6	0.00714
Non immobilized (NI)	18	8.5 ± 1.2	4.8 ± 1.9*	0.0287	7.1 ± 1.9	4.7 ± 1.4*	0.0179	46 ± 10	35 ± 9*	0.0169

*p* value statistically significant: VAS-b 24 m F vs. NF *p* = 0.0025, PF vs. NF *p* = 0.0042, I vs. NI *p* = 0.0043; VAS-l 24 m I vs. NI *p* ≥ 0.001; ODI 24 m F vs. NF *p* < 0.001, PF vs. NF *p* = 0.0067, I vs. NI *p* = 0.0076.

## 4 Discussion

**Figure 2:**
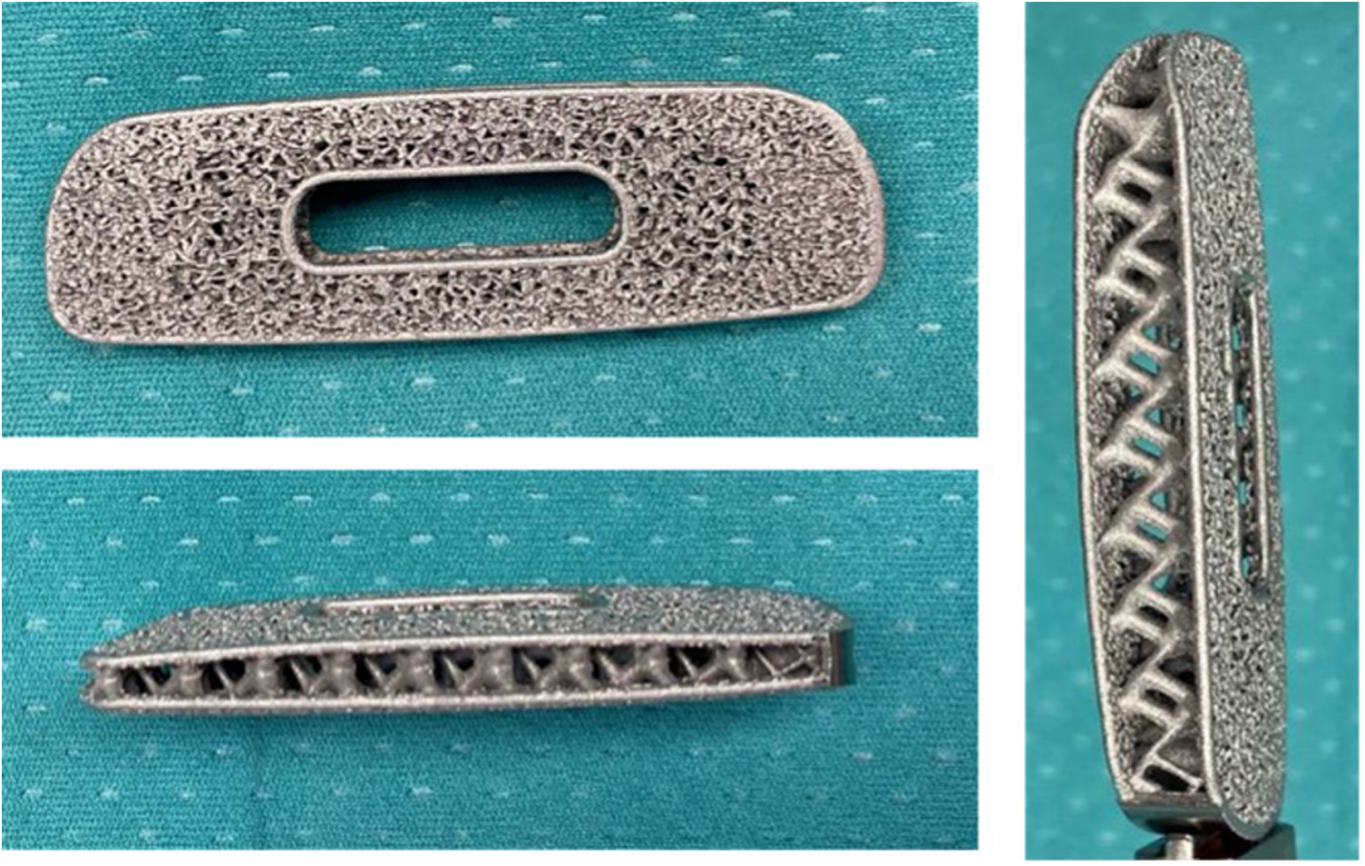
The porous titanium 3D printed cage offers a pathway for natural bone ingrowth promoting fusion.

**Figure 3:**
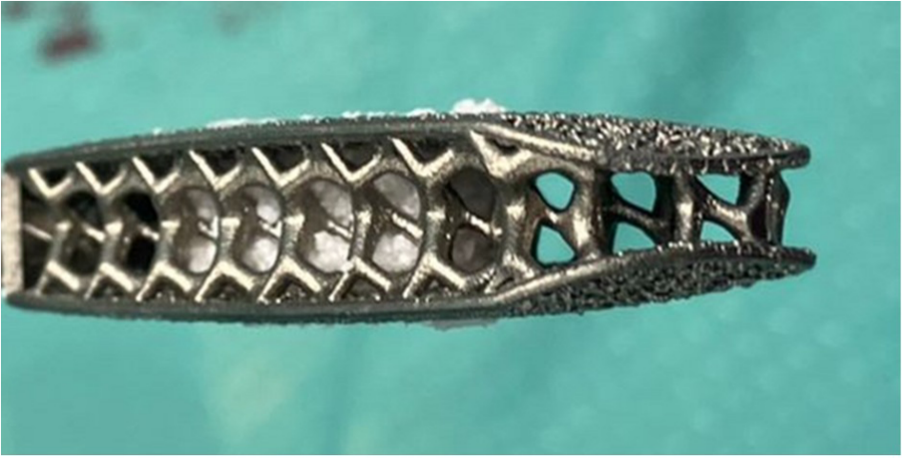
Example of how the cage is prepared before implantation, by placing synthetic bone (AttraX Putty Nuvasive, San Diego, CA, US) inside to facilitate interbody fusion.

Minimally invasive transpsoas LLIF is a safe and effective procedure for patients with various spinal conditions, including degenerative disc disease with mild-to-moderate central and/or foraminal stenosis, symptomatic spondylolisthesis, degenerative scoliosis, and adjacent segment failure. This approach has shown comparable postoperative clinical and radiographic outcomes to conventional open anterior or posterior lumbar interbody fusions (11–13). However, there is still no consensus on how to best evaluate segmental fusion. Different methods have been reported, focusing primarily on bone fusion between vertebrae, either inside or around the implants (14). Fusion is also critical for achieving segmental immobilization, which prevents micromovements that can cause mechanical stress and system failures, as well as persistent back pain and instability-related disorders. Evaluating immobilization is challenging, often relying on indirect radiological signs. While bone bridges (BB) may not suffice for segmental immobilization, meticulous disc space preparation is widely recognized as crucial for achieving fusion (15). Recent advancements in implant technology, particularly the development of 3D-printed porous titanium cages, have further improved LLIF outcomes. These cages are designed to mimic the porous structure of natural bone, promoting better osteointegration and stability. The biomechanical properties of these cages create an optimal environment for bone growth, leading to higher fusion rates and fewer instances of cage subsidence (16, 17). The use of instrumentation can significantly enhance fusion chances, with porous materials and 3D-printed titanium cages showing promise in reducing stress shielding and subsidence. Laboratory studies suggest that maximizing the bone-hardware interface area and creating implants with a texture and porosity of bone can minimize stress shielding and subsidence (11). Literature reports that porous materials reduce stress at the bone-hardware interface, leading to the development of a novel porous 3D-printed titanium interbody cage that allows better bone ingrowth on the cage surface. By 3D-printing titanium cages, rather than casting them, complex internal architectures and structural pores can be incorporated. This approach creates intricate internal geometries and a rough surface architecture, promoting increased osteoblastic activity and seamless integration into bone (18). Furthermore, Donaldson et al. (16) reported that the unique structural design of 3D-printed cages facilitates higher anterior and posterior fusion rates, which is critical for long-term stability and preventing mechanical complications, such as pseudoarthrosis, often a concern with conventional titanium cages (19, 20). For this reason, the use of porous titanium cages is becoming increasingly popular, as initial studies demonstrate a lower risk of subsidence. Kraft et al. (11) reported that subsidence of the vertebral cage occurred in 6.7% of all cases and in 3.4% of all lumbar levels, a lower rate compared to previously reported subsidence rates. Additionally, the porous architecture (Figures 2, 3) significantly reduces the risk of subsidence, a common issue with traditional implants that can lead to postoperative complications and the need for revision surgeries (16). The effectiveness of 3D-printed cages in LLIF is also evident in their ability to enhance patient outcomes. Recent studies highlighted not only the clinical efficacy but also the cost-effectiveness of using advanced materials and designs in spinal fusion surgeries (21). Furthermore, patient satisfaction rates post-LLIF using 3D-printed cages tend to be higher due to reduced pain and disability compared to those who undergo traditional fusion procedures (13).

In our cohort, we also observed a lower incidence of subsidence in the group with the novel porous cages (5.88%) compared to 9.88% in the group with conventional cages, with a statistically significant difference. These findings indicate that the architecture of porous titanium cages may facilitate favorable and faster bone ingrowth and bridging, contributing to successful spinal fusion and reducing the risk of implant subsidence. The results of this investigation revealed several important insights. Two years post-surgery, the group that received the novel porous titanium cages without posterior fusion exhibited a higher rate of intervertebral bone bridges (BB). In 17 segments (33.34%) of Group A, there was interbody fusion even without posterior fusion, which ensured mechanical stability. Conversely, in patients treated with the conventional titanium cage, we found 18 segments (21.42%) with partial fusion (absence of posterior fusion but presence of interbody fusion), a statistically significant difference compared to Group A. This is likely related to the better footprint of the titanium porous cage, which allows mechanical interbody fusion even without posterior fusion.

**Figure 4:**
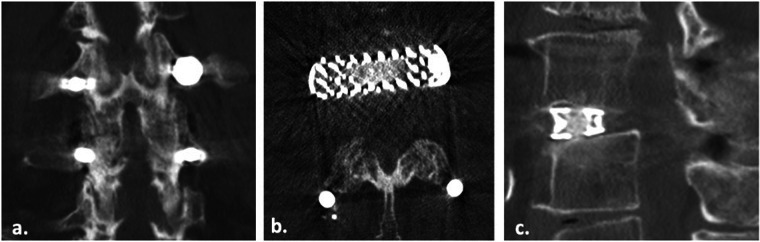
Coronal **(a)**, axial **(b)** and sagittal **(c)** CT scan images of a 72 years old patient showing complete fusion of zygapophyseal facet (Pathria Grade III) and interbody bony bridge (BB) fusion.

Additionally, the evaluation of zygapophyseal joints (ZJ) showed that posterior fusion was more prevalent in the porous cage group, with 92.15% of patients achieving grade III fusion, compared to 86.5% of patients treated with conventional titanium cages. This suggests that the use of porous titanium cages may be associated with better posterior fusion and consequently better segmental immobilization. The primary endpoint of solid fusion at 24 months was achieved in 172 segments (92.97% of total implanted cages) with the presence of BB and cage integration at the vertebral body interface, associated with posterior fusion of the facet joints (Figure 4). This fusion rate correlated with improvements in patient-reported disability, quality of life, and pain scores at 24 months compared to pre-surgery levels.

Although the role of spinal instrumentation and fusion continues to be debated in the treatment of adult spinal degeneration and deformity, our study demonstrates that 3D-printed porous titanium cages in LLIF surgery can achieve stabilization and fusion objectives. However, despite these advancements, the implementation of 3D-printed cages is not without challenges. The initial learning curve associated with these new technologies can result in complications, highlighting the importance of adequate training and experience for surgeons adopting this method (22). However, once the technology is mastered, the efficacy of porous cage designs in spinal fusion surgeries becomes evident. For instance, Fogel et al. (23) conducted a comprehensive mechanical and biological analysis of 3D-printed porous interbody cages, demonstrating that their microporous structure significantly reduces stress shielding and subsidence rates compared to conventional designs. This aligns with our findings where the novel porous cages showed a subsidence rate of 5.88%, significantly lower than the 9.88% observed with conventional cages. Further, Fogel et al. (24) highlighted that the material choice and structural design of fusion cages have a profound effect on osseointegration and subsidence performance. Their study demonstrated that porous titanium cages support enhanced bone ingrowth, leading to more robust fusion outcomes. This is consistent with the higher fusion rates observed in our study among patients treated with porous cages.

Additionally, biomechanical evaluations by Yee-Yanagishita et al. (25) provided evidence that modern porous cage designs outperform traditional cages in terms of subsidence resistance, especially in the lateral fusion approach. These findings further support our results, suggesting that porous titanium cages not only improve fusion but also contribute to better mechanical stability in LLIF procedures. Incorporating these findings into the broader context of implant design, it becomes evident that porous titanium cages offer significant advantages in achieving both anterior and posterior fusion, thereby enhancing clinical outcomes while reducing the risk of complications such as subsidence.

In conclusion, minimally invasive LLIF via a retroperitoneal transpsoas approach is a safe and effective method for treating various spinal diseases. Radiological evidence at the 2-year follow-up indicates successful interbody fusion, demonstrated by bridging bone and facet joint fusion. Our findings show that patients treated with the novel 3D-printed porous titanium cage (Group A) exhibit a higher incidence of interbody fusion in those without posterior fusion of the facet joints, and a lower incidence of subsidence. Further research and long-term studies are necessary to validate these findings and evaluate the long-term effects of using porous titanium cages in LLIF procedures.

## 5 Limitations

This study has a few limitations that need to be acknowledged. Firstly, the retrospective nature of the data collection might affect the level of evidence provided. Additionally, the radiological outcomes were based on images collected with an average follow-up period of 24 months post-surgery, which limits our ability to observe trends over time, despite having preoperative and 24-month postoperative CT scans and x-ray images.

## 6 Conclusions

While fusion rate is a commonly reported outcome after spinal fusion surgeries, the ultimate surgical goal is often segmental immobilization, which reduces mechanical stress on the instrumentation system and lowers the risk of failure. Our findings demonstrate that excellent fusion rates and segmental immobilization can be achieved in cases of adult degenerative disease and deformity using 3D-printed porous titanium cages. These excellent fusion rates also correlate with improved patient-reported outcomes. Further well-designed investigations are necessary to explore potential clinical–radiological correlations. The findings suggest that using 3D-printed porous titanium cages in LLIF procedures may offer a promising solution for achieving more reliable spinal fusion outcomes. The increased intervertebral bone bridges, improved posterior fusion rates, and reduced subsidence observed in the porous cage group underscore the potential benefits of this innovative technology in enhancing the fusion process. This study emphasizes the importance of implant design in promoting successful fusion and improving patient outcomes.

## Data Availability

The original contributions presented in the study are included in the article/Supplementary Material, further inquiries can be directed to the corresponding author.
